# Autogenous fresh demineralized tooth graft prepared at chairside for dental implant

**DOI:** 10.1186/s40902-015-0009-1

**Published:** 2015-02-18

**Authors:** Eun-Seok Kim

**Affiliations:** College of Dentistry, Dankook University, 126 Jukjoen-Dong Suji-Gu, Yongin-Si Gyeonggi-Do, 448 Korea

**Keywords:** Dental implant, Bone graft, Autogenous fresh demineralized tooth, Auto-FDT, Tooth osteoplantation

## Abstract

**Background:**

This study aimed to evaluate the clinical usefulness of autogenous fresh demineralized tooth (auto-FDT) graft prepared at the chairside for alveolar bone grafting during dental implant surgery.

**Methods:**

In total, 38 patients requiring both tooth extraction (for endodontic or periodontal reasons or third molar extraction) and alveolar bone regeneration for dental implant placement were included. Within 2 h after clean extraction, the teeth were prepared at the chairside to serve as bone graft material. In the same sitting, blocks or chips of this graft material were used to reconstruct defects at the osteotomy site simultaneously with or before implant placement. Twelve months after prosthesis fabrication and placement, the clinical findings and implant success rates were evaluated. Histological studies were randomly conducted for selected cases.

**Results:**

Clinical evaluation showed favorable wound healing with minimal complications and good bone support for the implants. No implant was lost after 12 months of function following prosthetic rehabilitation. Histological examination revealed new bone formation induced by the graft material.

**Conclusions:**

Chairside preparation of autogenous fresh demineralized teeth after extraction can be a useful alternative to the use of autogenous bone or other graft materials for the immediate reconstruction of alveolar bone defects to facilitate subsequent implant placement.

## Background

Although autogenous bone is considered the gold standard among graft materials, donor site morbidity may be an associated problem. Allogenic, xenogenic, and alloplastic graft materials have been used as alternatives, but they have a number of drawbacks compared with autologous grafts, such as decreased function, the potential risk of infectious disease, an unsatisfactory resorption pattern, a prolonged healing time, and high cost [1]. An ideal bone graft material should stabilize the blood clot; provide a biomechanical scaffold for cell migration, proliferation, and differentiation; contain functional proteins and peptides, such as growth factors; and exhibit appropriate resorption and remodeling during new bone formation. Materials based on collagen, particularly type I collagen, have attracted attention because of their ability to improve the cellular responses of osteogenic lineages, thus ensuring better bone regeneration [2]. However, the mineral component providing the biomechanical backbone remains an important factor for cell differentiation, nidus of calcification, and space maintenance during new bone formation [3]. Considering the hierarchical structure of bone, a mineralized collagen matrix with functional proteins and proper fibrillar arrays for biomechanics may be suitable as a natural bio-inspired graft material in bone tissue engineering [4].

The tooth is increasingly attracting attention as a material for alveolar bone regeneration. It is composed of an organic matrix and an inorganic reinforcing phase of hydroxyapatite. Radial arrays of dense type I collagen fibrils, which account for 90% of the organic matrix, and noncollagenous acidic proteins play an important role in calcification [5]. The chemical composition of dentin is very similar to that of bone. The inorganic content is 70%–75%, organic content 20%, and water content 10%. In alveolar bone, these components are present in proportions of 65%, 25%, and 10%, respectively [6]. However, tooth has much lower fat content and no marrow compared to bone, which make it easier to be changed into graft material [7]. A raw tooth cannot easily induce new bone formation because of its high mineral content, high crystallinity, and low porosity, which may interfere with migration, attachment, and proliferation of vascular and mesenchymal cells. The osteogenic capacity of a demineralized tooth was verified as early as 1967 [8], and it has been generally accepted that autogenous and allogenic demineralized teeth are osteoinductive or osteoconductive graft materials [9].

Commercial materials based on demineralized teeth have recently been used in human trials [1]. However, several shortcomings limit the popularity of these materials, including a long preparation time, limited productivity for commercial preparation, a consequent increase in cost, and dehydration that decreases the commercial shelf life. Some efforts to decrease the demineralization time did not satisfy the requirements for the simplicity of these materials and easy availability of different graft forms (block, chips, and powder) in the clinic [10].

This prospective study aimed to evaluate the clinical usefulness of autogenous fresh demineralized tooth (auto-FDT) graft prepared at the chairside immediately after extraction and used as a bone graft material for dental implant placement and to assess the potential of this material as an alternative for autologous bone and other graft materials.

## Methods

### Patient selection

This prospective clinical trial was performed at one center in Korea. The Institutional Review Boards (IRB) of Dankook University Jukjeon Dental Hospital in Yongin approved the study protocol (JDH2011-001). All patients provided written informed consent before treatment initiation. From February 2011 to August 2013, 38 consecutive patients who required tooth extraction for endodontic or periodontal reasons or pericoronitis of third molar and dental implant restoration were included in this study. The reasons for bone grafting included the repair of vertical or horizontal alveolar bone defects to facilitate implant osseointegration. Patients with generalized aggressive periodontitis, severe general illness (over ASA Class III) were excluded.

### Tooth extraction and preparation

Before surgery, all patients received intramuscular administrations of clindamycin phosphate (300 mg) and tramadol hydrochloride (50 mg). Local anesthesia was induced by nerve block or local infiltration using 2% lidocaine HCl (1:80,000 epinephrine) and additional bupivacaine HCl if required. Following clean tooth extraction, any soft tissue adherent to the root was removed using a surgical blade. The pulp tissue in the root canal(s) was then removed using a pear-shaped carbide bur (no. 330), and several small holes 2–3 mm apart for better contact to reagent were created from the outer surface of the tooth to pulp chamber.

### Chairside preparation of the auto-FDT graft

The entire chairside preparation process, including demineralization, sterilization, and washing was completed within 2 h (block type) after extraction according to the manufacturer’s instructions using an ultrasonic device and reagents (VacuaSonic® and DecalSi-DM®; Cosmobiomedicare, Seoul, Korea). The final washing solutions for the processed teeth were sent to the Green Cross Clinical Laboratory (Yongin, Korea) for bacterial culture for monitoring.

### Auto-FDT grafting and implant placement

**Figure 1:**
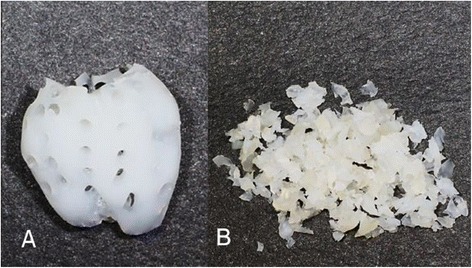
** The demineralized tooth was processed to either blocks or chips. A**. Autogenous fresh demineralized tooth (auto-FDT) blocks. **B**. Auto-FDT chips.

The fresh extraction sockets were prepared for bone grafting. The auto-FDT graft was implanted as blocks or chips depending on the condition of the defect (Figure 1). The block was trimmed with a surgical blade or bone rongeur, while the chips and powder were prepared using a bone mill or crusher. The dental implant fixtures (TSII-CA, Osstem, Seoul, Korea) were placed simultaneously with or after graft placement. An absorbable or titanium sheet barrier membrane was used to cover the graft. Patients were prescribed antibiotics and non-steroidal anti-inflammatory drugs for 1 week and were given postoperative dental hygiene instructions.

### Evaluation of new bone formation and implant stability

**Figure 2:**
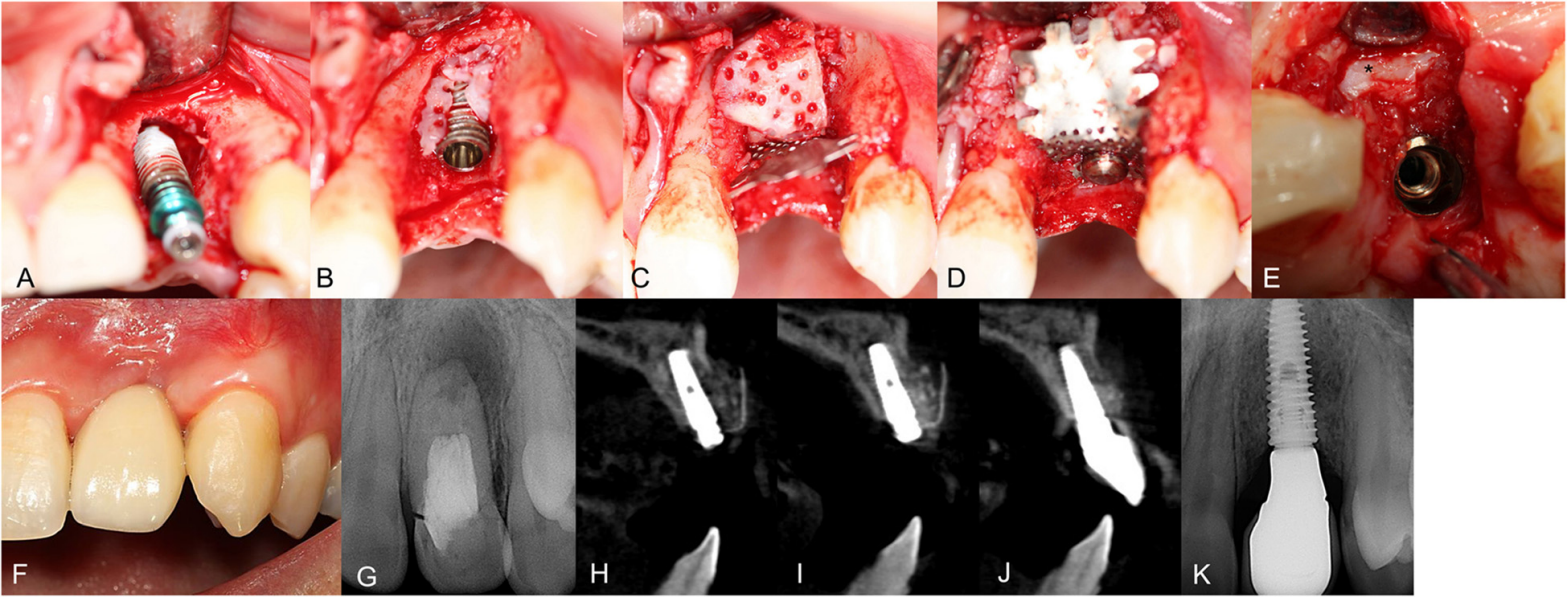
**Clinical case of autogenous fresh demineralized tooth (auto-FDT) graft to support dental implant placement in a 23-year-old male. A**. A large labial dehiscence remains after fixture placement in the socket of the maxillary left lateral incisor. **B**. The processed tooth chips are grafted for intimate contact with the fixture. **C**. The entire defect is covered with the block. **D**. A thin titanium sheet (CTi-mem™, Neobiotech, Seoul, Korea) is used to hold the graft. **E**. An uncovering procedure is performed 5 months after grafting. The tooth block is observed tightly fixed to the host bone. **F**. Five months after grafting, the final prosthesis is placed. **G**. A periapical radiograph shows the periodontal and periapical lesions around the lateral incisor. **H–J**: Series of cone beam computed tomography images. Immediately after grafting **(H)**. After 5 months, an increase in radiopacity and decrease in volume of the graft indicate bone remodeling **(I)**. Twelve months after final prosthesis placement, the regenerated bone provides good maintenance and support for the implant **(J)**. Intraoral radiography obtained 12 months after final prosthesis placement. **K**. Intraoral radiography obtained 12 months after final prosthesis placement.

Postoperative wound healing and implant stability were clinically assessed. Radiographs, including panoramic views and cone beam computed tomography (CBCT) images, were obtained to confirm graft healing at 6 month postinsertion and 12 months after prosthesis delivery (postoperative 18 months). Implant stability was measured using a radio frequency device (Osstell Mentor, Integration Diagnostics, Göteborg, Sweden) before recording impressions. Prosthetic procedures were initiated 4–6 months after fixture placement depending on the type of defect and graft amount. The radio frequency value was measured twice in two directions (buccal and lingual or palatal; Figures 2A–K).

In some cases, small bone segments were acquired from the graft site of the osteotomy with a trephine bur during fixture placement or the graft over cover screws with the tissue punch during uncovering. Decalcified tissue sections were then prepared using routine procedures and stained with hematoxylin–eosin (H&E) and Masson’s trichrome (MT).

### In-vitro assessment of the auto-FDT graft

Dried sample weighing and energy dispersive X-ray spectroscopy (EDS) were used to evaluate the degree of tooth demineralization. Extracted maxillary premolars (n = 5) were dried and their weight was measured. The teeth were then processed in an ultrasonic chamber as for clinical use, and their dry weight were measured again. EDS (TEAM System, EDAX Inc., NJ, USA) was used to check the relative calcium content (wt%) at 5 points up to a depth of 700 μm from the surface of the dentin. Data were collected in the 2θ range of 8°–90°, with a step size of 0.02° and a counting time of 20 s at each step. The data bank from the International Center for Diffraction Data was used in a search/match program for phase identification.

Scanning electron microscope (SEM) studies were performed using the Nanoscope IIIa instrument (Digital Instruments, Tonawanda, NY, USA) after coating samples with a gold–palladium alloy. The processed tooth materials and untreated tooth surfaces were then observed.

### Statistical analysis

Wilcoxon signed rank test was used for in-vitro analysis of calcium using SPSS 20 software (IBM SPSS Inc., Chicago, IL, USA) with the significant level of 0.05, and the clinical data are described using descriptive statistics.

## Results

### Clinical observations

In total, 38 patients (29 men and nine women; mean age, 49.8 years; range, 21–79 years) were included in this study. All patients were followed up for 12 months after final prosthesis placement. A total of 58 teeth were extracted and processed. None of the graft materials showed bacterial growth on perioperative culture testing.

The defects at the osteotomy site requiring implant placement were restored with graft material prepared using the teeth from the original extraction sites (n = 30) or third molars (n = 8). Horizontal and vertical augmentations were performed in 31 patients and sinus augmentation (lateral approach) in seven. A total of 58 implants were placed, with the mandibular posterior region being the most common site (26 implants). The implants ranged from 3.5 to 4.5 mm in diameter and 7 to 13 mm in length.

The graft materials were covered with a collagen membrane in 29 patients and a titanium mesh in nine. Eight patients received implants 3–6 months after bone grafting. All patients exhibited uneventful healing. No significant complications were observed, such as compromised wound healing, uncontrolled dehiscence, local wound infection, graft failure, and dental implant failure. Four patients showed minor wound dehiscence in the early stage of soft tissue healing; however, complete healing by secondary intention was observed within 4 weeks.

Three to 6 months after grafting or final prosthesis placement, increased radiopacity with homogeneity was observed on panoramic radiographs, while CBCT images confirmed good implant support (Figure 2). The mean Implant Stability Quotient (ISQ) at the time of final prosthesis fabrication was 72.7 ± 5.2 (68–81), which was a clinically acceptable range. No case of implant failure was observed during the follow up period.

**Figure 3:**
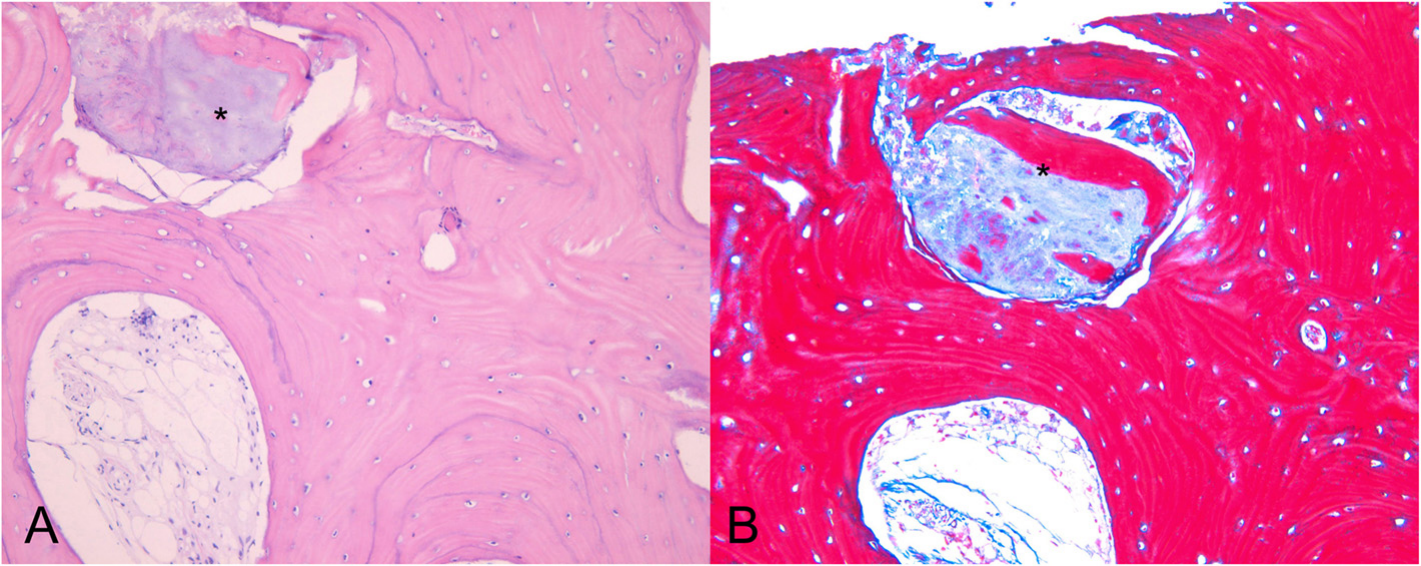
**Histologic examination revealed new bone remodeling. A**. Histological sections at 5 months after surgery show remodeling of new bone around the autogenous fresh demineralized tooth (auto-FDT) graft (H&E staining, ×200). Some auto-FDT fragments show resorption signs (asterisk). **B**. Fusion-like integration between two matrices of the graft and new bone at the interface can be observed (asterisk; Masson’s trichrome staining, ×200).

Histological examination of guided bone regeneration revealed good to excellent new bone formation with an even spatial pattern within 3–6 months after grafting. Remodeling and maturation with osteocytes contained within lacunae were observed. Some of the auto-FDT material had integrated strongly with the newly formed bone, while some showed resorption and became less visible. There was no sign of chronic inflammation or a granulomatous reaction (Figures 3A, B).

### In-vitro assessment of the auto-FDT graft

EDS confirmed significant changes in the calcium and phosphorus (mineral components of the tooth) and carbon, nitrogen, and oxygen (elements of the organic matrix) levels after treatment; 81.1% calcium was removed (from 31.59 ± 1.81 to 5.98 ± 2.47 wt%), while 46.6% dry weight was lost during processing.

**Figure 4:**
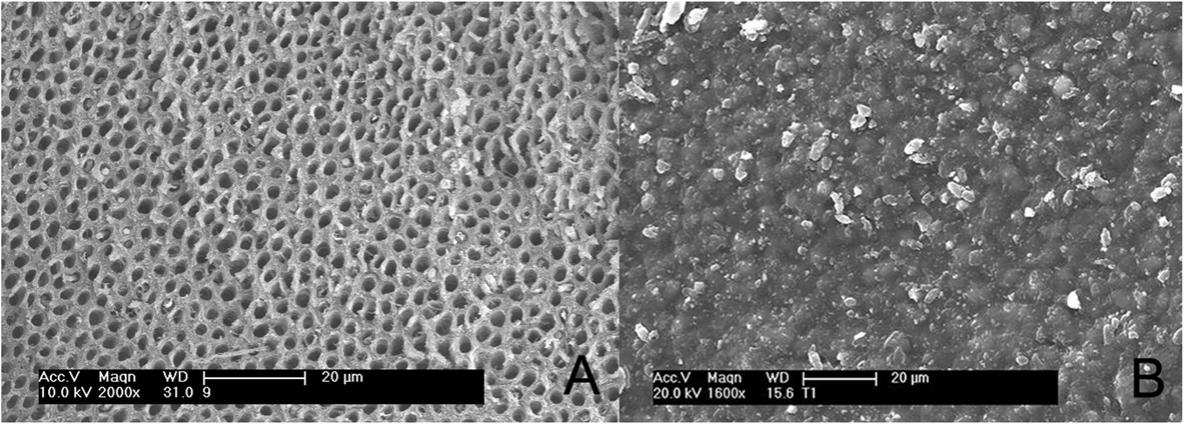
**SEM images showed surface difference between treated and untreated teeth. A**. A scanning electron micrograph of the surface of an autogenous fresh demineralized tooth (auto-FDT) graft (scale bar, 20 μm). The dentinal tubule size has increased after processing’. **B**. A scanning electron micrograph of an untreated dentin surface (scale bar, 20 μm). The smear layer is composed of small particles of dentin and dendrites covering the dentin surface.

On the surface of the auto-FDT graft, SEM observations revealed the absence of enamel and cementum and the presence of distinct dentinal tubules surrounded by a dense collagen matrix. The dentinal tubules on the surface of the graft material were wider than those observed in untreated teeth (Figures 4A, B).

## Discussion

According to search of the Pubmed database, the first use of demineralized dentin for bone regeneration in humans dates back to 1975; in that case, an allogenic source was used [11], whereas an autogenous source was first used only in 2003 [12]. The present study is likely to be the first human trial of auto-FDT prepared at the chairside and implanted on the day of extraction. Placement of a tooth into an alveolar socket after treatment is called “tooth replantation” or “tooth transplantation”. Auto-FDT grafting for alveolar bone regeneration can be referred to as a “tooth osteoplantation”. Demineralization is a crucial step in tooth grafting. Although a nondemineralized form of tooth powder showed good results [13], demineralization of bone and teeth increases the bioavailability of matrix-associated noncollagenous proteins such as osteocalcin, osteonectin, bone sialoprotein, phosphophoryn, and bone morphogenetic protein; these may enhance new bone formation [14-16]. Considering the higher mineralization and crystallinity of the tooth compared with those of bone, a decrease in mineral components and crystallinity may be more important for tooth-based graft materials [17]. Tooth demineralization is time consuming (usually 2–6 days), thus limiting the use of FDT as a graft material. Nevertheless, FDT has shown great potential in alveolar bone regeneration [18,19]. Another drawback of demineralization is that prolonged acid exposure may negatively affect noncollagenous proteins involved in new bone formation [20]. Murata et al. reported a device and method to decrease the demineralization time for tooth graft material, but this method only provides the powder type and uses an organic acid, which is not recommended for processing. In addition, these authors did not describe a specific final sterilization method for the graft material [10].

To overcome these limitations, we adopted a modified ultrasonic technology. The preliminary tests revealed that a regular ultrasonic cleaner does not dramatically decrease the processing time because the tooth is comprised of numerous microsized dentinal tubules surrounded by peritubular dentin, which is more sclerotic than intertubular dentin [21]. Periodic negative pressure can eliminate ultrasonic pocketing and the implosion loss of cavitation, which decrease the efficacy of ultrasound, and allows deep penetration of the cavitation energy and reagents into the dentinal tubules [22,23]. A thermoelectric cooler prevents the temperature increase caused by strong ultrasonic power and maintains the temperature under 40°C. These advances facilitate quicker demineralization and prevent thermal damage to the tooth proteins. The short duration of the entire process enables grafting on the same day of extraction and also increases the clinical availability of auto-FDT, because the block form of the graft can be trimmed or easily converted into chips or powder depending on the condition of the bone defect during surgery.

Implant stability was confirmed by a radio frequency device and histological examination in this study. The ISQ of all implants was satisfactory for final prosthesis placement. Histological examination revealed appositional bone growth around the auto-FDT graft, and its resorption implies that the demineralized tooth was osteoconductive with a remodeling characteristic. The interesting histological features observed in this study included the appearance of fusion-like integration between two matries of the graft and new bone at the interface with an undistinguishable border and new bone formation in the auto-FDT graft (Figure 3). This implies that dentinal tubules provide niches for cells involved in osteogenesis. Possible reasons for the good results in this study include the high biocompatibility of autogenous tissue, its collagen-based porous structure that is beneficial for cell function, minimal changes in the structure of mineralized collagen without dehydration (freeze drying), and rehydration procedures and preservation of beneficial noncollagenous proteins involved in mineralization. This study strongly supports the view that auto-FDT grafts provide a useful biological scaffold comprising three-dimensional macro- and microarchitecture with osteopromotive osteoconduction suitable for new bone formation [24].

## Conclusions

The use of auto-FDT prepared at the chairside as bone graft material on the day of tooth extraction can be convenient for both patients and clinicians. Within the limitations of this study, the results indicate that auto-FDT graft prepared within 2 h of extraction can effectively restore alveolar bone defects in humans. We conclude that the availability of auto-FDT graft would have a strong positive effect on the clinical use of the tooth as a bone graft material in implant surgery. Further studies should be performed to confirm the osteogenic effects and biological safety of this tooth-based graft material.

## Abbreviations

auto-FDT: Autogenous fresh demineralized tooth graft
CBCT: Cone beam computed tomography
H&E: Hematoxylin–eosin
MT: Masson’s trichrome
EDS: Energy dispersive X-ray spectroscopy
SEM: Scanning electron microscope
GBR: Guided bone regeneration
ISQ: Implant stability quotient
